# New Species of Empidinae (Diptera) from San Rossore National Park, Italy, with the First Report on Leg Polymorphism in the Genus *Hilara* Meigen and Their DNA Barcoding Evidence †

**DOI:** 10.3390/insects16010083

**Published:** 2025-01-15

**Authors:** Miroslav Barták, Milan Kozánek, Antonio Belcari, Andrea Š. Tóthová

**Affiliations:** 1 Department of Zoology and Fisheries, Faculty of Agrobiology, Food and Natural Resources, Czech University of Life Sciences Prague, Kamýcká 129, 165 00 Praha-Suchdol, Czech Republic; bartak@af.czu.cz; 2 VirNat s.r.o., Na medzi 1C, 83106 Bratislava, Slovakia; milan@virnat.sk; 3 Department of Agriculture, Food, Environment and Forestry (DAGRI), University of Florence, Piazzale delle Cascine 18, 50144 Firenze, Italy; antonio.belcari@unifi.it; 4 Department of Botany and Zoology, Faculty of Science, Masaryk University, Kotlářská 2, 611 37 Brno, Czech Republic

**Keywords:** *Empis*, *Rhamphomyia*, Palaearctic region, Europe

## Abstract

Altogether three species of Empidinae are described from San Rossore National Park, Italy: *Empis* (*Euempis*) *sanrossorensis* Barták sp. nov., *Hilara polymorpha* Barták sp. nov., and *Rhamphomyia* (*Megacyttarus*) *sanrossorensis* Barták sp. nov. Polymorphism in the shape of foreleg in *Hilara* is reported for the first time. The COI sequences for barcoding purposes and upcoming studies are provided.

## Simple Summary

The effective management and appreciation of protected natural areas requires a thorough understanding of the intricate animal communities that have developed over time. A central goal of this complex study is to identify the diverse species of animals, plants, and microorganisms that constitute this complex biological community. This is particularly important for insects, which, due to their small size and biological characteristics, pose challenges in sampling and classification. This research contributes to a larger project launched in 2021 that aims to study the true fly fauna (Diptera) of the San Rossore Natural Park (Tuscany, Italy). As a result of extensive sampling, three species new to science are described and illustrated.

## 1. Introduction

Coastal ecosystems are situated at the interface between marine and terrestrial sedimentary environments and are prone to frequent alterations in their morphological structure and vegetation landscape [1]. These ecosystems exhibit significant biodiversity in terms of plant species and communities, often along a well-defined gradient, provided they have not undergone excessive modification [2]. San Rossore National Park is located within a highly urbanized region, experiencing a substantial influx of visitors throughout the year. Despite the negative impact of human pressure, it has retained considerable natural features. Its diverse ecosystems are rich in flora and fauna. As a result of its ecological value, San Rossore National Park was designated a UNESCO Biosphere Reserve in 2004.

A diversity of habitats provides favourable conditions for the occurrence of abundant and species-rich dipteran communities. Several dipterists have conducted research in San Rossore National Park. Extensive studies of Dolichopodidae [3], Tabanidae [4], Sciomyzidae [5], Chaoboridae [6], and records of various species from the families Opomyzidae, Lauxaniidae, Drosophilidae, Tephritidae, Psilidae, Sciomyzidae, Dolichopodidae, and Empididae [7] demonstrate the unique nature of the dipteran fauna in San Rossore, which remains largely unexplored. In 2021 and 2022, extensive collections were conducted to study the dipteran fauna of San Rossore in detail.

Empididae is a large family of Diptera Brachycera with about 1300 species known from the Palaearctic region and almost 1000 of them belonging to the three genera: *Empis* Linnaeus, 1758, *Hilara* Meigen, 1822, and *Rhamphomyia* Meigen, 1822. The Empididae primarily comprises predators capturing prey (usually small arthropods) on the ground, water surface or in flight. Representatives of the subfamily Empidinae also visit flowers and in some environments represent important pollinators (e.g., in Arctic regions or in high mountains). Larvae are predaceous and are found in soil, wood, aquatic, and semiaquatic environments. Adults are well known for their mating behaviour, including swarms with nuptial gift transfer by males to females.

Concerning the DNA analyses, even if we do not attempt to resolve phylogenetic relationships, we consider the DNA evidence of newly described species as of high importance. With *Empis* (*Euempis*), the females are not well processed, e.g., they are not known in the most likely closest species, *E. azrouensis*, hence DNA tools may be very useful to prove correct association of males and females. In the case of *Hilara,* it constitutes the very first published data on the polymorphism of the shape of an otherwise diagnostically very important and, in other species, completely constant character, such as the shape and pubescence of the front tibia and basitarsus, so we wanted to track its impact on the DNA level.

## 2. Material and Methods

### 2.1. Study Area

The San Rossore Estate is situated between the Serchio and Arno rivers in the province of Pisa. Administratively, it is part of the regional state property and is managed by the Migliarino-San Rossore-Massaciuccoli Regional Park. Established in 1979, it protects approximately 23,150 hectares of dunes, mesophilous, and xerophilous forests, wetlands, and agro-forestry landscapes, along approximately 30 kilometres of coastline.

Recognizing its exceptional landscape and biological value, the San Rossore Estate is included in the UNESCO World Network of Biosphere Reserves as part of the “Coastal Forests of Tuscany”. The interplay of natural processes and human activities has resulted in a diverse range of vegetation types, including several priority habitats. The floristic inventory includes over 600 plant species [8].

### 2.2. Sampling Methods

**Figure 1:**
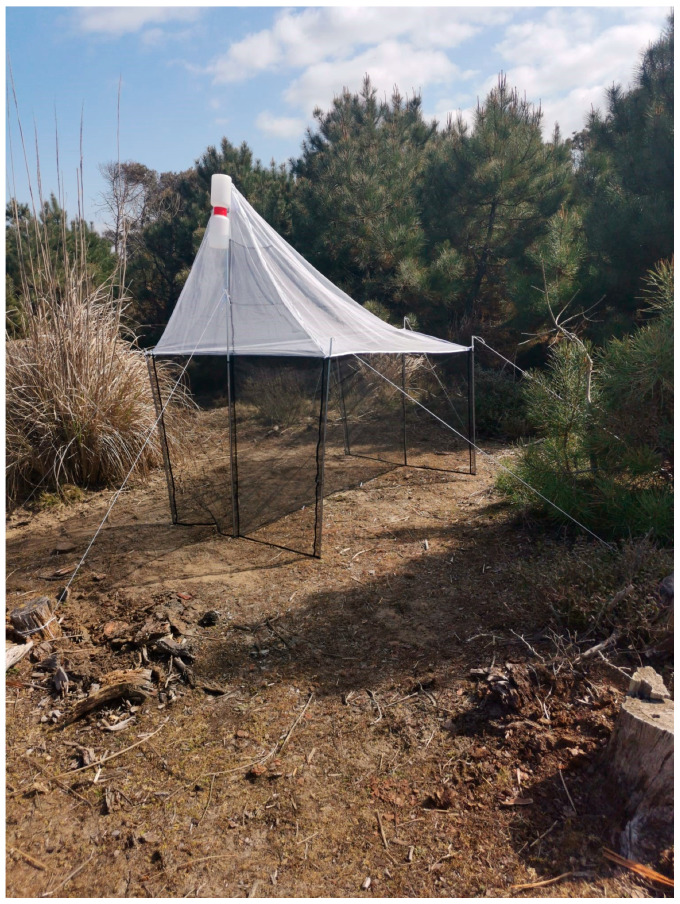
Seashore.

**Figure 2:**
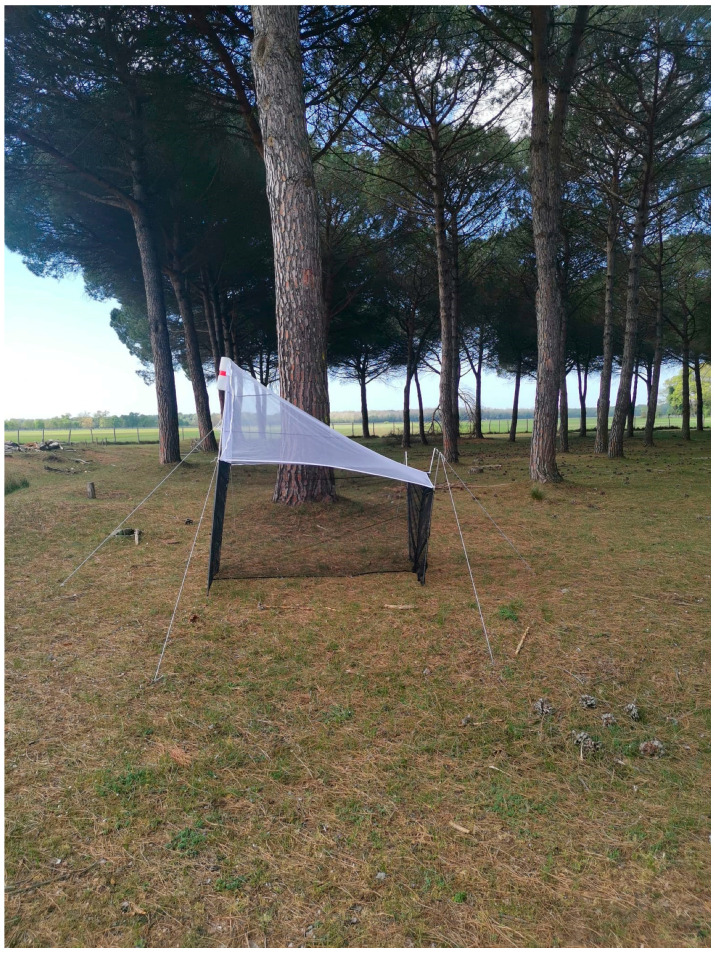
*Pinus* grove.

**Figure 3:**
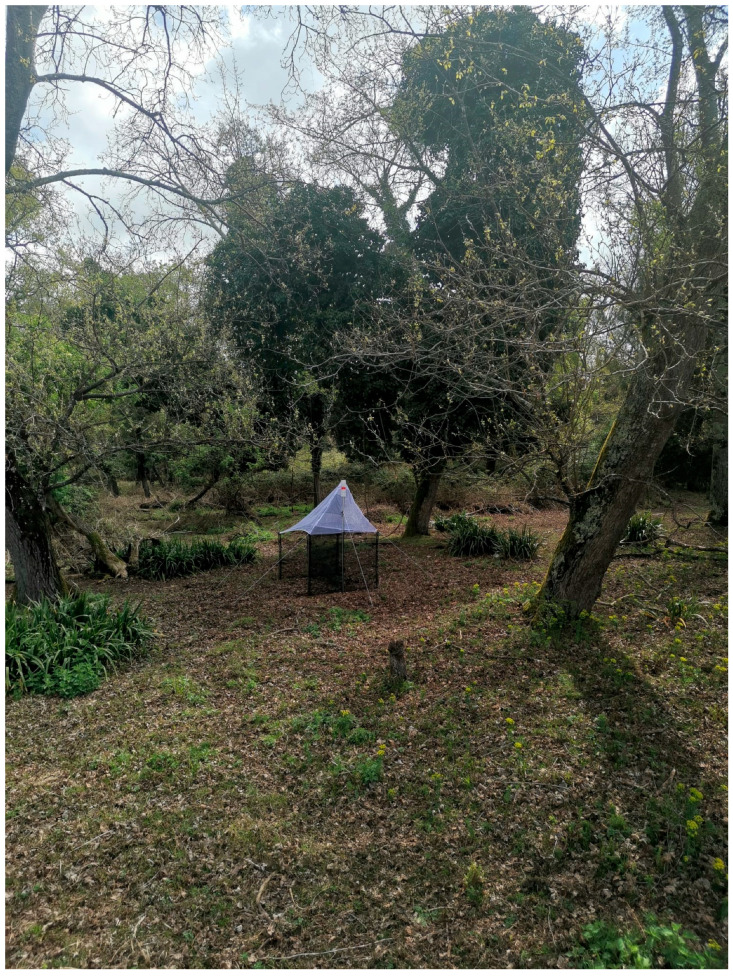
Forest.

Sampling was conducted using Malaise traps (MT) during the collection seasons of 2021 (from April 6 to December 18) and 2022 (from May 5 to December 1). Three traps were installed each year. The collecting sites were: (1) seashore (Figure 1) consisting mainly of calcareous sand and bordered by dunes where several herbaceous species and sclerophylous shrubs grow, such as *Agropyron junceum* (L.) P. Beauv., *Ammophila arenaria* (L.) Link, *Sporobolus pungens* (Schreb.) Kunth, *Helycrisum stoechas* (L.) Moench and further internally, some small *Pinus pinea* L. born from seed, (2) xerophilous pine forest-conifer plantations of anthropogenic origin (*Pinus* grove on labels) (Figure 2) with *Pinus pinaster* Aiton and *Pinus pinea*, and (3) meso-hygrophilous–broad-leaved forest (Figure 3) with dominated *Quercus robur* L., *Fraxinus angustifolia* ssp. *oxycarpa* (M. Bieb. Ex Willd.) mixed with *Carpinus betulus* L., *Populus alba* L. and *Alnus glutinosa* (L.) Gaertn.

### 2.3. Material Treatment

From large materials of Empididae stored in 70% ethylalcohol, only voucher specimens were selected and dried using methods described by [9]. Remaining materials were stored in ethylalcohol for DNA studies. The morphological terms follow [10,11,12]. All body measurements (including body and setae length) were taken from dry specimens (therefore the actual length may differ from that of fresh or wet-preserved material) by means of an ocular micrometre mounted on a Nikon SMZ 1500 binocular microscope. Male body length was measured from the antennal base to the tip of genitalia and female body length from the base of the antennae to the tip of the cerci. Thoracic setae are counted on one side of the body except scutellars. Geographical coordinates in decimal format are used. Material depository: Czech University of Life Sciences Prague (CULSP).

### 2.4. DNA Extraction, Amplification, Sequencing

For the DNA analyses, two specimens of both *Hilara polymorpha* sp. nov. and *Empis sanrossorenis* sp. nov. (male and female) were chosen. We included the different leg types of *H. polymorpha* (discussed below) to discover the impact of this morphological feature on the COI sequence differences. DNA extraction and amplification was provided by the Molecular Biology and Genetics Laboratory of the Slovak National Museum-Natural History Museum, in Bratislava, Slovakia. For DNA extraction, a piece of insect tissue stored in 96% ethanol was provided. Ethanol was removed and the tissue was dried in Concentrator plus/Vacufuge^®^plus (Eppendorf AG, Hamburg, Germany) at 30 °C, 15 min. Homogenization of tissue was achieved by Tissue Homogenizer, FastPrep^®^-24-5-G (MP Biomedicals, LLC, Santa Ana, CA, USA). Genomic DNA was extracted using the DNeasy^®^Blood and Tissue Kit (Qiagen, Hilden, Germany) according to the manufacturer’s protocol. The region of mitochondrial cytochrome c oxidase subunit I genes (CO1) was selected as a target for DNA amplification with barcoding primers LCO1490 (5′ GGTCAACAAATCATAAAGATATTGG 3′) and HCO2198 (5′ TAAACTTCAGGGTGACCAAAAAAT 3′) [13]. The total quantity of 13–15 ng of template DNA was added to the PCR reaction mix. The PCR reaction was performed by the GoTaq^®^ G2 DNA Polymerase kit (Promega, USA) using a BioRad C1000 Touch™ Thermal Cycler. The partial gene was sequenced in a commercial laboratory (Eurofins Genomics GmbH, Cologne, Germany). The obtained sequences were deposited in GenBank.

### 2.5. DNA Analyses

The obtained barcoding sequences of the COI gene marker were compared to chosen available taxa from GenBank (see Table 1). The COI fragments were aligned according to amino acid translations using online MAFFT v. 7 [14] on the MAFFT server (http://mafft.cbrc.jp/alignment/server/, accessed on 10 December 2024). The dataset consisted of 25 ingroup and 2 outgroup taxa.

The genetic distances of the barcoding region of COI of the analyzed taxa of Empididae (+Dolichopodidae as outgroup) were calculated in MEGA version 11 [15] using the Kimura 2-parameter (K2P) model and are presented in Table 2 and Table 3.

The resulting phylogram reflecting the interspecific relations was conducted using MrBayes v. 3.2.7 [16] on the CIPRES computer cluster. The node support values are given with the posterior probability (PP) above the nodes (if value >0.5) and the bootstrap value (BV) below the nodes in the resulting tree… For the BI, 5 million generations were conducted and the convergence of the runs was assessed by checking the potential scale reduction factor (PSRF) values of each parameter (in all cases, approaching 1.000) and the standard deviation of split frequencies (<0.01). The mean log-likelihood value for the best-fit BI tree was −5016.56). The resulting tree was visualized using iTOL [17].

## 3. Description of New Species

**Figure 4:**
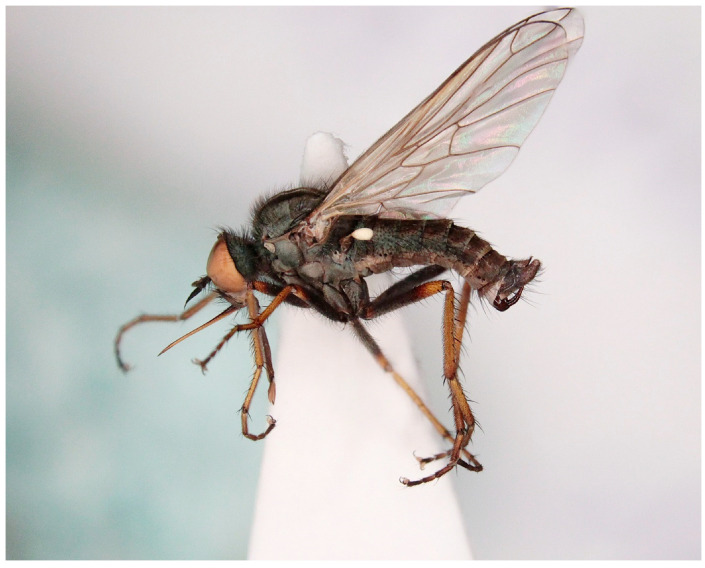
*Empis* (*E.*) *sanrossorensis* Barták sp. nov., paratype male, habitus.

**Figure 5:**
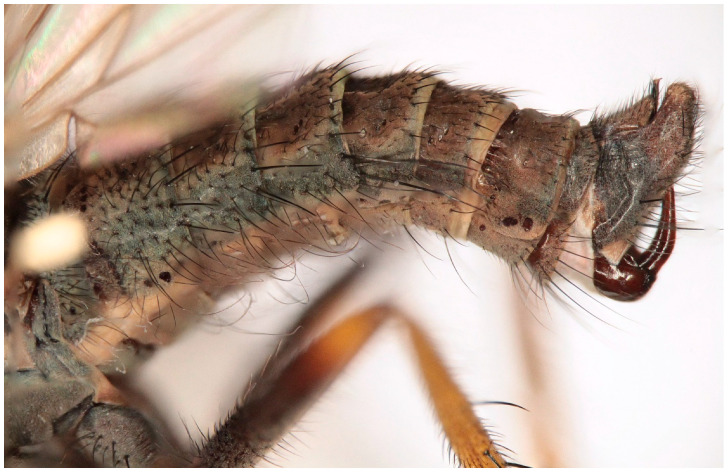
*Empis* (*E.*) *sanrossorensis* Barták sp. nov., paratype male abdomen.

**Figure 6:**
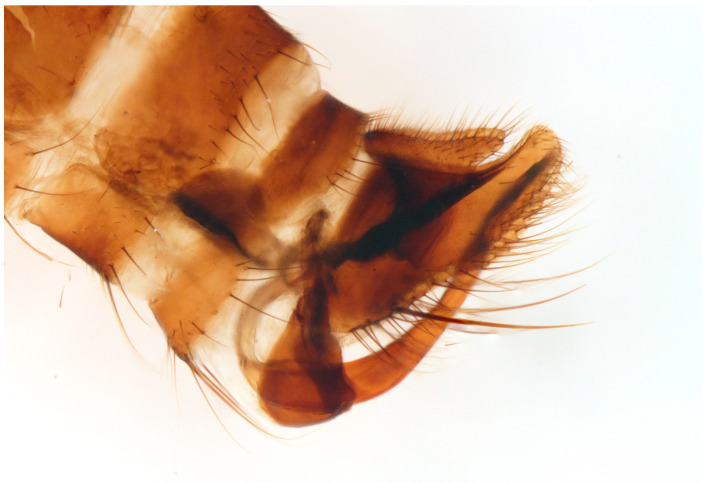
*Empis* (*E.*) *sanrossorensis* Barták sp. nov., paratype male genitalia, macerated.

*Empis (Euempis) sanrossorensis* Barták sp. nov.

(Figure 4, Figure 5 and Figure 6)

Type material: HOLOTYPE ♂, Italy: San Rossore NP, Pisa prov., forest, 43.725, 10.313 MT, A. Belcari, 6-16.iv.2021, deposited in CULSP. PARATYPES: The same data as for holotype, three males, two females; same locality but 27.iv.-7.v.2021 one male, one female; San Rossore NP, Pinus grove, 43.695, 10.341, MT, A. Belcari, 21-31.viii.2021, two males, four females; San Rossore NP, Pisa prov. forest + seashore, 43.7, 10.3 MT, A. Belcari, 7-17.v.1922, one male. All the type material is pinned and deposited in CULSP.

Diagnosis: Middle-sized species, entirely black setose; palpus brown; mesoscutum almost uniformly covered with fine setae. Large setae: notopleurals and supralars; acrostichal and dorsocentral setae multiserial; legs with at least tibiae yellow (in male) or entirely yellow (in female); halter yellow; male abdominal segments with strong and elongated posteromarginal setae laterally on tergite 4 but without shiny spots or conspicuous outgrowths; epandrial lamella narrowed apically, with one to three long strong setae; cercus simple.

Description: Male. Head: Black, rather light grey microtrichose, holoptic (eyes meeting over long distance). Frons (small triangle just above antennae and much smaller below front ocellus) without setae. Dorsal half of eye with distinctly larger facets than ventral half. Ocellar setae fine, black, half as long as frons and ocellar triangle with several additional fine setae half as long. Occiput densely setose in mid and lower parts (setae as long as or even longer than ocellars). Face about 0.35 mm broad ventrally, microtrichose, without setae. Clypeus shiny dorsally, gena very narrow. Palpus brown, with long (0.30 mm distally up to 0.40 mm proximally) black setae. Labrum brown, lustrous, nearly twice head height. Antenna black, both basal segments long setose; length of antennal segments (scape: pedicel: postpedicel: basal joint of stylus: last joint of stylus) = 0.13–0.15 mm: 0.10–0.11 mm: 0.39–0.43 mm: 0.02–0.03 mm: 0.14–0.17 mm, respectively. Thorax: black, light grey microtrichose, entirely black setose; mesoscutum with three blackish brown stripes down rows of acrostichal and dorsocentral setae. Both anterior and posterior spiracles pale. Chaetotaxy: pronotum with irregular row of black setae (about as long as occipitals), prosternum, proepisternum, and propleura with numerous fine setae; acrostichals multiserial (not arranged in rows but some 4–6 setae across rows, anteriorly only narrowly separated from dorsocentrals) and about 0.20 mm long; dorsocentrals sub-equally long and multiserial, ending in several irregularly arranged prescutellars; sides of mesoscutum densely setose with setae similar to dorsocentrals, even prescutellar area with several inclinate setae; stronger setae (postpronotal, presutural intra- or supra-alars and prealars) not differentiated from fine setae; 1–2 supraalars; notopleuron with irregular row of 5–7 strong setae posteriorly and fine additional setae; one very long and strong postalar; two pairs of long scutellars; laterotergite with black setae. Coxae black, similar in colour to pleura, femora mostly brown except yellowish tips and bases, tibiae and proximal parts of tarsi yellow, distal parts of tarsi darkened. Fore femur anteriorly with bare stripe, anteroventrally with setae as long as femur depth, posteriorly and posteroventrally with setae up to twice as long as femur depth in distal part, shorter in proximal part. Fore tibia with a row of 8–10 rather strong anterodorsal setae about as long as tibia depth and somewhat finer, more numerous and slightly longer posterodorsals; ventrally very short and fine setose. Mid femur dorsally and anteroventrally with fine setae shorter than femur depth and a row of posteroventrals about as long as femur depth, apically with several longer and finer setae. Mid tibia with several (4–6) antero- and posteroventral and anterodorsal setae being slightly longer than tibia depth and with 2–3 posterodorsal setae only in basal half of tibia up to three times tibia depth. Hind femur short setose dorsally except several longer and stronger dorsal setae on apical part and basally, posterior row of rather strong setae as long as femur depth, posteroventrally short and fine setose, anteroventrally with a row of setae up to as long as femur depth apically, shorter basally. Hind tibia with short ventral ciliation, anterodorsally with 6–8 setae as long as tibia depth and posterodorsally with similar row of setae nearly twice as long. Fore basitarsus with several setae dorsally slightly longer that basitarsus depth; mid basitarsus dorsally short setose, ventrally with several somewhat longer setae and hind basitarsus dorsally with setae up to twice basitarsus depth, following hind tarsomeres with rather long dorsal and preapical setae. Comb at tip of hind tibia with long and strong seta. Wing membrane colourless; veins yellowish brown; axillary angle strongly acute; costal seta long; CuA + CuP complete. Halter yellow, calypter whitish with yellow margin and black fringes. Abdomen: brownish black, light grey microtrichose, black setose. Lateral marginal setae on tergites 2–4 longer than respective segments, very long on segment 4, segments 5–7 with progressively shorter setae; discal setae on dorsum of abdomen very short; venter with posteromarginal setae as long as segments, discal setae short, sternite 1 bare. Genitalia (Figure 6): hypandrium small, bare; epandrium boat-shaped, with one to three long setae, cercus simple and small, phallus narrowed apically. Length: body 6.0–6.1 mm, wing 5.5–5.9 mm.

Female: Dichoptic, all facets equal in size, frons about 0.30 mm broad with about 10 long black setae on each side. Thoracic chaetotaxy as in male but all body setae shorter than in male. Legs yellow including tarsi, last tarsal segment darkened, coxae black as in male, similarly setose as in male, only setae shorter. Exceptions: posterodorsal setae on mid tibia much shorter than in male (about as long as tibia depth), hind tibia with distinct setae also ventrally. Abdomen with sternite 8 lustrous, other parts grey microtrichose; posteromarginal setae are on tergites 2–4 shorter than respective segments, on following segments very short (about as long as discal ones). Length: body 7.1–7.4 mm, wing 6.1–6.9 mm.

Etymology: The species epithet is an adjective derived from the name of the Italian Natural Park San Rossore near Pisa, where this species was collected.

Remarks: The new species resembles *Empis* (*E.*) *dasycera* (Collin, 1960), *Empis* (*E.*) *picipes* Meigen, 1804, and *Empis* (*E.*) *pleurica* (Collin, 1960). All four species have modified (long and strong) posteromarginal setae on sides of abdominal tergite 4 (Figure 5). In the male of *E. picipes* (distributed mostly in temperate central regions of Europe, Greece, Italy, and north-western part of European Russia [18]) and *E. dasycera* (Jordan, Israel, Turkey [18]), the posterior lateral corner of abdominal tergite 4 is produced and bears some strong setae ([19] p. 402, Figure 3; [20] p. 509, Figure 187; [21] p. 28, Figure 19). In the male of *E. pleurica* (Croatia, Iran, Israel, Russia (North Caucasus), Turkey [22]), abdominal tergite 4 is not produced posterolaterally but, contrastingly with the new species, tergite 4 has a more or less distinct shiny patch on each side and tergite 5 is shiny black and convex laterally. In the new species, tergite 4 has neither a shiny spot nor produced margin, only modified long and strong setae. Considering its general appearance (Figure 4) and especially the male genitalia (Figure 6), the new species is similar to *Empis* (*E.*) *azrouensis* Shamshev, 2022, however it is larger (body longer than 6 mm in the new species but 4.3 mm in *E. azrouensis*), its hind tibiae do not have anteroventral setae (one seta in *E. azrouensis*), and there are slight differences in the genitalia (basal part of phallus narrower and apical part slightly bent in the new species, broader in basal part and straight apically in *E. azrouensis*), but most striking is the modified setosity of tergite 4 (unremarkable in *E. azrouensis*)

The barcoding sequences are deposited in GenBank under access numbers PQ344943 (male) and PQ344944 (female).

**Figure 7:**
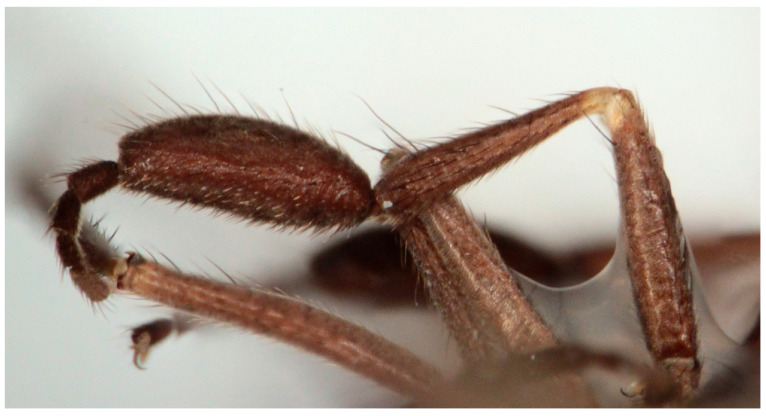
*Hilara polymorpha* Barták sp. nov., paratype male fore leg type I.

**Figure 8:**
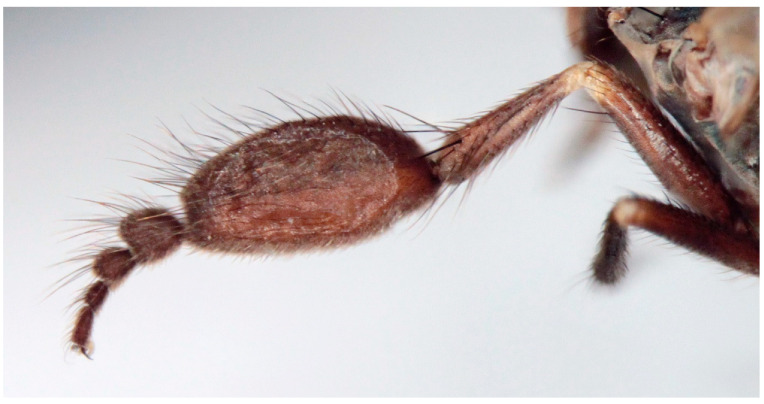
*Hilara polymorpha* Barták sp. nov., paratype male fore leg type II.

**Figure 9:**
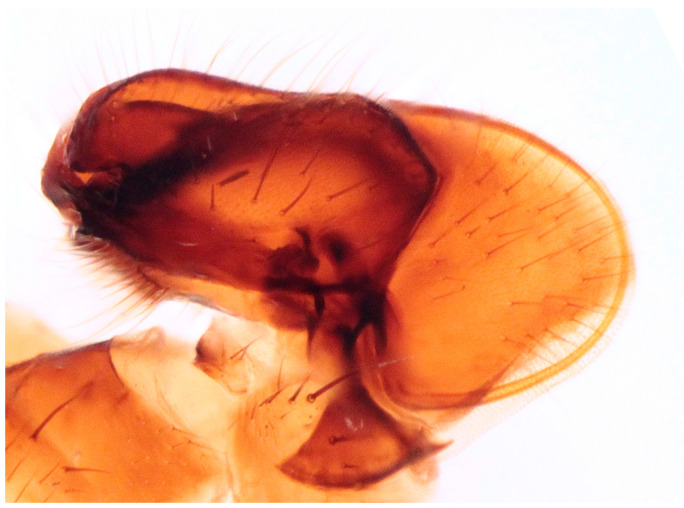
*Hilara polymorpha* Barták sp. nov., paratype male genitalia, macerated.

**Figure 10:**
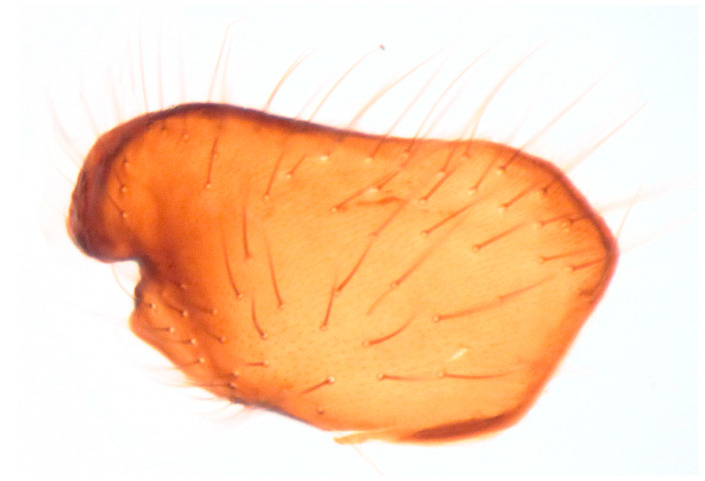
*Hilara polymorpha* Barták sp. nov., paratype male epandrium (left lamella).

**Figure 11:**
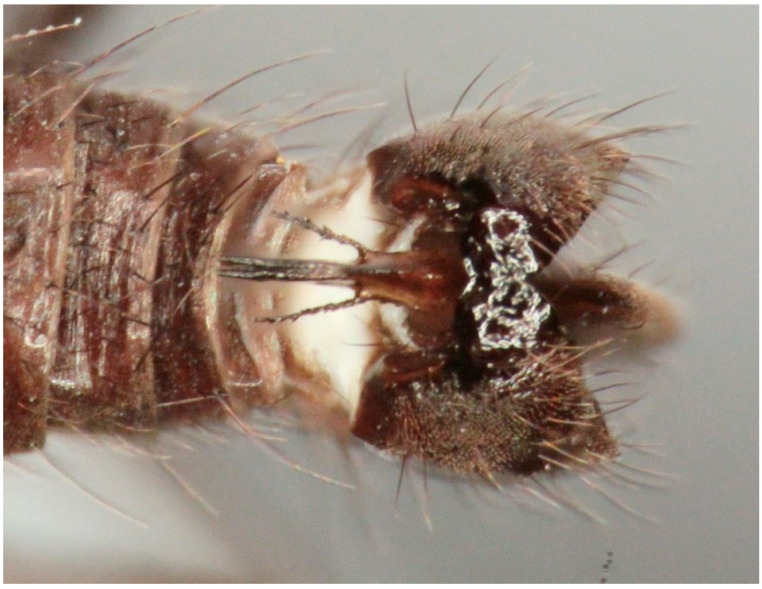
*Hilara polymorpha* Barták sp. nov., paratype male genitalia, dorsal view.

**Figure 12:**
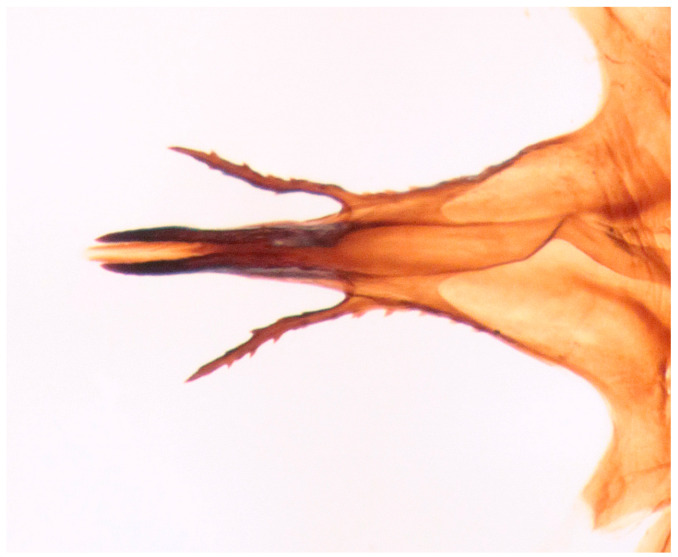
*Hilara polymorpha* Barták sp. nov., tip of hypandrium of paratype male, dorsal view.

*Hilara polymorpha* Barták sp. nov.

(Figure 7, Figure 8, Figure 9, Figure 10, Figure 11 and Figure 12)

Type material: HOLOTYPE ♂, Italy: San Rossore NP, Pisa prov. seashore, 43.723, 10.280 MT, A. Belcari, 16-27.iv.2021 (type I fore leg, deposited in CULSP). PARATYPES: type I foreleg: same data as for holotype, 17 males; same locality but 6-16.iv.2021 4 males; San Rossore NP, Pinus grove, 43.695, 10.341, MT, A. Belcari, 27.iv.-7.v.2021, 2 males; same locality but 6.-14.vi.2021, 2 males; same locality but 21.-31.viii.2021, 1 male; San Rossore NP, Pisa prov. forest, 43.725, 10.313 MT, A. Belcari, 6-16.iv.2021, 6 males; same locality but 17-27.v.2021, 2 males; same locality but 27.iv.-7.v.2021, 3 males. Type II foreleg: same data as for holotype, 12 males; same locality but 6.-16.iv.2021, 8 males; San Rossore NP, Pisa prov. forest, 43.725, 10.313 MT, A. Belcari, 27.iv.-7.v.2021, 2 males; San Rossore NP, Pinus grove, 43.695, 10.341, MT, A. Belcari, 27.iv.-7.v.2021, 3 males; same locality but 6-16.vi.2021 3 males. Females: same data as for holotype, 22 females; same locality but 6-16.iv.2021 2 females; San Rossore NP, Pinus grove, 43.695, 10.341, MT, A. Belcari, 16-27.iv.2021, 1 female; same locality but 27.iv.-7.v.2021, 3 females; same locality but 17-27.v.2021, 1 female; same locality but 6.-14.vi.2021, 1 female; San Rossore NP, Pisa prov. forest, 43.725, 10.313 MT, A. Belcari, 6-16.iv.2021, 2 females; same locality but 27.iv.-7.v.2021, 6 females; same locality but 17-27.v.2021, 2 females. All the type material is pinned and deposited in CULSP.

Distribution: Pisa province, Italy.

Diagnosis: Middle-sized black species of *Hilara* with black legs, halter, and occiput, quadriserial acrostichals and mesoscutum with three dark brown stripes on lines of setae. Male hypandrium produced with long finger-like lateral projections near apex. Male forelegs polymorphic, specimens differing especially in the ratio of length of tibia and basitarsus, shape of basal joints of fore tarsus and chaetotaxy as described below. Female hind tibia slightly swollen, flattened and curved.

Male. Head: dichoptic, all facets approximately of same size. Head black, occiput deep velvety black in dorsal view, dark brownish grey microtrichose in posterior view. Frons broad (0.10 mm above antennae and 0.20 mm at level of front ocellus), velvety black with lighter grey triangle reaching from antennal bases to about two thirds, with one pair of frontal setae (0.20 mm long) and several additional setae on sides. Ocellar setae black, about 0.25 mm long, ocellar triangle with several additional short hair-like setae. Face rather light grey, about 0.11 mm broad at middle, slightly widening ventrally, bare. Occiput in dorsal half with only postocular row of setae slightly shorter than frontal pair, distinctly incurved and second row of several setae half as long, in ventral half irregularly and sparsely covered with fine black setae, postocular row complete. Clypeus lustrous, genae microtrichose. Antennae black, ratio of antennal segments (scape: pedicel: postpedicel: basal segment of stylus: apical bare mechanoreceptor) = 0.06–0.07 mm: 0.06–0.07 mm: 0.22–0.24 mm: 0.15–0.17 mm: 0.02–0.03 mm, respectively), both basal antennomeres rather short setose. Labrum brown, shiny, two thirds as long as head height, labellae broad and rather long setose. Palpus brown, short, with several short setae and long subapical seta. Thorax: brownish black, light grey microtrichose, mesoscutum with broad brown stripe below acrostichals and narrower brown stripes below dorsocentrals. All setae black, only proepisternum with very short pale setulae. Chaetotaxy: prosternum bare, proepisternal depression with 1–2 setulae; antepronotum with a single long and strong black seta on each side; postpronotum with one longer seta and several much shorter setulae; fine irregularly quadriserial acrostichals (about 15 setae in a row), about 0.10–0.15 mm long, some specimens with almost biserial acrostichals in anterior part; dorsocentrals uniserial and slightly longer than acrostichals, ending in 1–3 times longer and stronger prescutellars; presutural area nearly bare except 0–1 dorsocentral seta outside row, one fine well differentiated presutural intraalar and one long and strong presutural supraalar; two black notopleurals (anterior part of notopleuron with several very small black setulae); 1–2 short supraalar and several praealar setae in a row; one long postalar; four scutellars (two pairs, outer pair shorter). Legs brown with yellow knees, microtrichose, black setose. Coxae brownish black, black setose. Seta in comb at tip of hind tibia absent. Fore femur with short setae, posterodorsally and posteriorly with elongate setae about as long as femur depth. Forelegs of two different types: type I with basitarsus about as long as tibia, about three times as long as broad and dorsally with only several setae shorter than basitarsus depth, fore tarsi not or very slightly swollen, dorsally with ordinary setulae, tibia with elongate fine setae posterodorsally (slightly longer than tibia depth) and with 3–4 preapical long and strong setae (Figure 7); type II with basitarsus about 1.5 times as long as tibia, nearly twice as long as broad, dorsally rather densely covered with setae slightly shorter than basitarsus depth, tarsal joints 2–3 swollen, almost as broad as long, dorsally with long setae, also remaining tarsomeres often rather long setose dorsally, tibia with similar posterodorsal setae as in type I but with more numerous (up to 10) strong preapicals (Figure 8). Mid femur with complete row of long and strong anterior setae, other setae short. Mid tibia short setose, without stronger setae, posteriorly with elongate fine ciliation being slightly longer than tibia depth. Hind femur with short setose, anteroventral setae somewhat longer in apical third. Hind tibia anteroventrally with several setae slightly longer than tibia depth, dorsally short setose except long preapical anterodorsal seta. Mid and hind tarsi unremarkable, basitarsi thin and short setose. Wing clear, veins yellowish brown, CuA + CuP incomplete. Discal medial cell elongated, longer than vein M_2_. Costal seta long and black, axillary angle slightly obtuse. Halter brown, calypter dirty yellow with slightly darkened lateral margin and yellow fringes. Abdomen: brown, tergites sublustrous, venter microtrichose. Setae mostly black, sometimes first tergites on sides with paler setosity. Sternite 1 bare. Posteromarginal setae on sides of tergites about as long as respective segments, discal setae shorter than marginals; sternites almost bare, with only indistinct small setulae. Terminalia (Figure 9, Figure 10, Figure 11 and Figure 12) enlarged, with produced hypandrium; dorsal process of epandrium short and broad, lustrous (Figure 11); tip of hypandrium with two serrate long lateral processes very visible in dorsal view (Figure 11 and Figure 12). Length of body 2.7–3.8 mm, wing 3.3–4.1 mm.

Female. Similar to male. Femora as in male, tibiae short setose, without remarkable setae. Hind tibia slightly swollen, flattened and curved. Abdomen shorter setose, posteromarginal setae shorter than respective segments, sternite 8 slightly produced, cerci slightly broadened. Length of body 3.0–3.5 mm, wing 2.8–3.7 mm.

Etymology: The species epithet is an adjective reflecting the polymorphism of male forelegs (from New Latin “polymorphus” meaning “multiform shape”).

Remarks: The species described above is distinctive due to its pronounced polymorphism in the shape of the fore tibia and basitarsus. In most *Hilara* species, the shape and bristling of the foreleg are considered crucial species-specific characteristics. Roach [23] studied the North American species of *Hilara* and gave his results in his PhD thesis in 1971. The results of this revision were never published. The author mentioned a species under an MS name, which reflects the existence of different morphs (“*dimorpha*”). Males of *H. polymorpha* with type II legs resemble *H. balearica* Chvála, 2008, characterized by greatly swollen and long setose first three fore tarsomeres. Both types share a similarly produced hypandrium and mesoscutum with three dark stripes. However, they differ in several significant features: acrostichals are quadriserial (biserial in *H. balearica*); the costal seta is very long (very small in *H. balearica*); the halter is blackish brown (dirty yellow in *H. balearica*); and the legs have different setosity, particularly the fore tibia with long, fine posterior and posteroventral setae and a circlet of long setae preapically (short setose in *H. balearica*). The mid femur has a complete row of long and strong black anterior setae (fine brownish in *H. balearica*), and the abdomen is mostly black setose (including genitalia) with strong posteromarginal setae (pale setose with hind marginal bristles scarcely differentiated in *H. balearica*). Several specimens exhibited intermediate characteristics between the two types of forelegs, primarily in the ratio of tibia and basitarsus length. We selected the holotype from males with type I legs because this type was significantly more common (we estimate that type II represented less than 5 percent of the total unselected material).

The barcoding sequences are deposited in GenBank under access numbers PQ344941 (leg type I) and PQ344942 (leg type II). Sequences of both specimens are similar, thus indicating that the leg types are in the scope of intraspecific variability.

**Figure 13:**
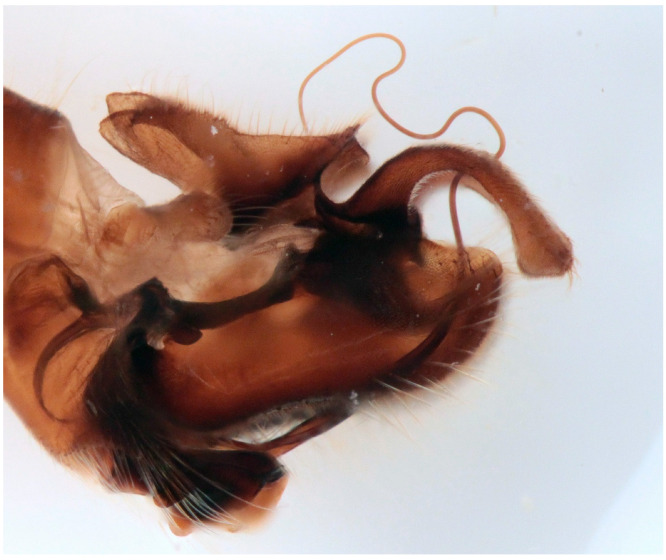
*Rhamphomyia sanrossorensis* Barták sp. nov., paratype male genitalia.

**Figure 14:**
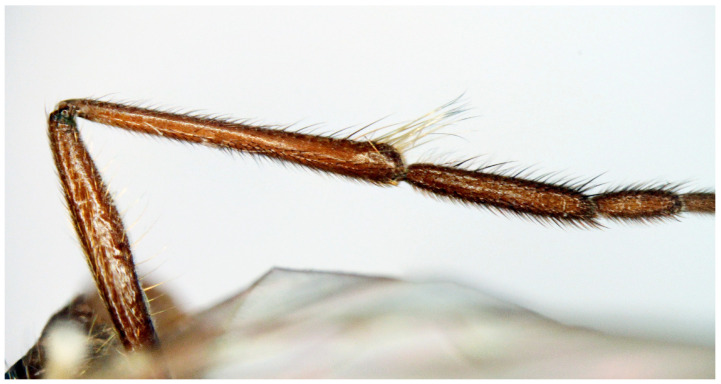
*Rhamphomyia sanrossorensis* Barták sp. nov., paratype male hind femur and basitarsus.

**Figure 15:**
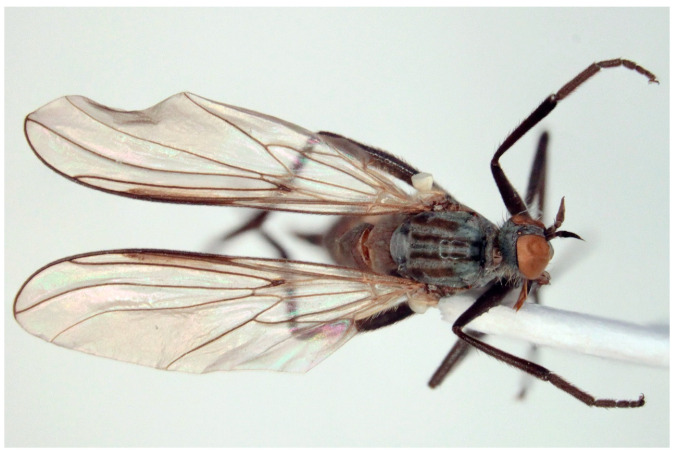
*Rhamphomyia sanrossorensis* Barták sp. nov., paratype female.

*Rhamphomyia (Megacyttarus) sanrossorensis* Barták sp. nov.

(Figure 13, Figure 14 and Figure 15)

Type material: HOLOTYPE ♂, Italy:, San Rossore NP, Pisa prov. forest, 43.725, 10.313 MT, A. Belcari, 6-16.iv.2021, deposited in CULSP. PARATYPES: same data as holotype, four males, two females; same locality but 27. iv.-7.v.2021 one male, one female; San Rossore NP, Pinus grove, 43.695, 10.341, MT, A. Belcari, 21-31.viii.2021, two males (CULSP).

Distribution: Pisa province, Italy.

Diagnosis: Rather large black species of *Rhamphomyia* subgenus *Megacyttarus*, being very similar to *R. crassirostris* (Fallén, 1816) in male (phallus with two loops, both fore and mid basitarsi narrow with short setae) but female with quite different wing venation (without closed discal medial cell and without any trace of vein M_2_).

Male. Head: dichoptic, all facets approximately of same size. Head including frons, face, occiput, clypeus, and very narrow genae black, light grey microtrichose. Frons slightly broader than front ocellus, slightly widening ventrally, with two rows of 6–8 setae up to 0.20 mm long. Ocellar setae black, fine, about as long as frons (0.40 mm), ocellar triangle with several additional short setae. Face broader than frons, widening ventrally, bare. Occiput sparsely covered with short black setae, some longer white setae ventrally, postocular row complete but irregular. Antennae black, length of antennal segments (scape: pedicel: postpedicel: stylus including basal segment) = 0.18–0.20 mm: 0.08–0.11 mm: 0.38–0.42 mm: 0.11–0.13 mm, respectively. Both basal antennomeres rather short setose (longest setae about 0.15 mm long). Labrum brown, shiny, as long as or slightly longer than head height. Palpus brown, short, with several short setae. Thorax: black, light grey microtrichose, mesoscutum with two sharp blackish brown stripes between acrostichals and dorsocentrals and similar much broader stripes outside of dorsocentrals; mostly light grey stripes below acrostichals and dorsocentrals are somewhat obscured, not very light grey but rather brownish grey, especially in anterodorsal view. Large setae including acrostichals and dorsocentrals black, fine setae outside of dorsocentrals, on front part of notopleuron and proepisternum yellowish white. Laterotergite with fan of yellow setae. Chaetotaxy: proepisternum with several rather long white setae; prosternum and proepisternal depression bare; antepronotum with usual collar of setae (black, laterally yellow); postpronotum with a single long set and several shorter pale setae; fine biserial diverging acrostichals about 0.15 mm long; dorsocentrals slightly longer than acrostichals, irregularly biserial and divergent, ending mostly in several longer and stronger prescutellars; presutural intraalar not clearly differentiated from fine setae on their areas; presutural supraalar usually stronger, yellow to black; 2–4 black notopleurals (anterior part of notopleuron with rather long and fine white setae); 1–2 supraalar and 0–3 praealar setae; 1 long black and several small white postalars; 2–4 scutellars (1–2 pairs) and further 2–4 shorter setae. Legs, including coxae, brownish black, microtrichose, both black and yellow setose. Very short seta in comb at tip of hind tibia. Fore femur with short setae dorsally, anteroventrally with pale setae about as long as femur depth, almost bare posteroventrally, except several setae on basal part. Fore tibia with short and fine homogeneous ventral setation, dorsally with setae shorter than tibia depth, only several anterodorsal setae slightly longer. Mid femur with anteroventral setae about as long as femur depth (ending in short rather spinose black setae), posteroventrally with incomplete row of longer setae, all mostly yellow. Mid tibia with all setae shorter than tibia depth, only several posterodorsals slightly longer. Hind femur with very distinct posterior tubercle, dorsally short setose, ventrally with mostly yellow setae sub-equally long as femur depth. Hind tibia (Figure 14) anteroventrally in apical part with dense homogeneous setation about as long as tibia depth and with prepical tuft of some ten long, mostly yellow setae, all other setae short. Fore basitarsus short setose, mid basitarsus thin and short setose, with short ventral spines; hind basitarsus narrow, dorsally with several setae slightly longer than its depth. Wing very slightly brownish, stigma darker, veins brown, CuA + CuP depigmented, vanishing before tip. Costal seta very small to indistinct, axillary angle right. Halter pale yellow, calypter yellow with yellow fringes. Abdomen: brownish black, venter light grey microtrichose, tergites in dorsal view dark blackish brown. Setae nearly all pale (whitish yellow to yellow) except sometimes several black hind marginals on tergites. Sternite 1 bare. Posteromarginal setae on sides of tergite 1 long, on tergite 2 as long as this segment, on remaining tergites slightly shorter, discal setae slightly shorter than marginals; sternites with sparse and somewhat longer setae on posterior margin. Terminalia (Figure 13) very similar to nearly all other members of *R. crassirostris* group of the subgenus *Megacyttarus*, differing from *R. crassirostris* mainly by medially incised hypandrium. Length of body 5.2–5.8 mm, wing 5.8–6.5 mm.

Female. Dichoptic, facets subequal in diameter. Frons about 0.20 mm wide, parallel-sided. Thorax as in male, light grey colour under acrostichals and dorsocentrals more sharply differentiated from brown stripes under setae. Fore femur with irregularly arranged anterior to anteroventral pale setae as long as or slightly longer than femur depth. Fore and mid tibia short setose. Mid femur dorsally and posteroventrally with very short mostly black setae, anteroventrally almost bare. Hind femur and tibia very short setose. Basitarsi of all legs thin and very short setose. Wing (Figure 15) without discal medial cell. Abdomen brown, light grey microtrichose, with very short setae (except somewhat longer on basal two tergites). All setae white except several dark on last segments including cerci. Length of body 6.1–6.8 mm, wing 5.9–6.4 mm.

Etymology: The species epithet is an adjective derived from the name of the Italian Natural Park San Rossore near Pisa, where this species was collected.

Remarks: Male *Rhamphomyia sanrossorensis* Barták sp. nov. is a member of the *R. crassirostris* group of the subgenus *Megacyttarus* (as delimited by [24]) superficially very similar to male *Rhamphomyia* (*M.*) *crassirostris,* having two loops on phallus and narrow and short setose fore and mid basitarsi. However, it may be separated by the tip of the tibia having a tuft of about 10 mostly yellow setae, which are much longer than dorsal setae on hind basitarsus (smaller number of mostly black setae similarly long as dorsal setae on hind basitarsus in *R. crassirostris*) and prominent posterior tubercle on hind femur (similar to that in *Rhamphomyia* (*M.*) *poissoni* Trehen, 1966, only very indistinct in *R. crassirostris*). There is a small difference in the genitalia: the ventral margin of hypandrium is incised medially (best visible in lateral view) in the newly described species and simply curved in *R. crassirostris*. Females have quite different wing venation without discal medial cell and without any trace of the vein M_2_. This character occurs among female *Megacyttarus* only in nearctic *Rhamphomyia* (*M.*) *rhytmica* Barták, 2002 belonging to quite different species group (and sometimes, as monstrosity, in other species, but in this case mostly with at least a stub of vein M2). Moreover, female fore femora have long irregularly arranged setae anteroventrally (about as long or longer as femur depth) but only very short setae in *R. crassirostris*.

## 4. Discussion

The European (or West Palaearctic) species of Empididae are relatively well studied, compared with other regions; however, even here there are recently described new species ([18,25,26,27,28,29,30,31]).

**Figure 16:**
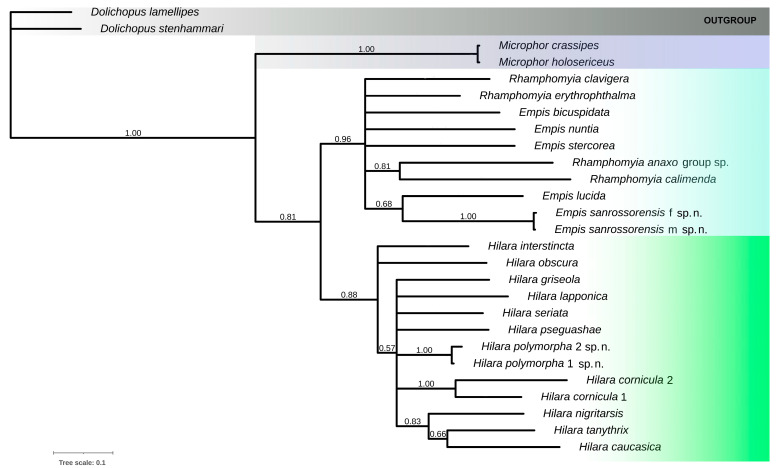
Bayesian hypothesis for the analyzed taxa based on 657 bp of COI barcoding sequence, the PP >50 are shown above the branches.

To contribute to the complex knowledge of biosystematics, we decided to also include the barcoding sequences of the two new species. The BI analysis based on COI sequences resulted in a phylogram depicted in Figure 16. Between the samples (leg type I and II) of the same species of *H. polymorpha* sp. nov., the pairwise distance was 0.09%, so this morphological feature has no taxonomic value.

Leg polymorphism is a frequent phenomenon, particularly evident in species exhibiting pronounced sexual dimorphism in leg morphology. This phenomenon may be associated with sexual mosaicism. For instance, intersex individuals of the genus *Fannia* often possess a male (or intermediate) head, male genitalia (frequently underdeveloped), and legs with characteristics intermediate between those of males and females [32]. Daugeron et al. [33] reported asymmetry in the shape of the fore tarsus in an *Empis* species. They documented not only males displaying an asymmetrical shape but also males with both tarsi narrowed and both tarsi broadened. However, our extensive material collection revealed no males with asymmetrical forelegs. Furthermore, in our study, we observed not only strict dimorphism but also intermediate cases, all of which displayed symmetrical leg morphology. Additionally, males with narrow tarsi (and comparatively longer tibiae) were significantly more prevalent in our samples compared to males with extremely broad tarsi and relatively shorter tibiae.

The *Hilara* fore basitarsus contains silk-producing glands [34]. However, the swollen basitarsus could be a male secondary sexual character in itself, with or without a glandular silk-producing function [35]. Here, we describe a species with a markedly different form of this tarsomere, which may affect male mating success. Deeper studies may help to resolve this problem in the future.

According to the COI fragment analyses, we discovered a rather high inter-specific distance amongst both *Hilara* and *Empis* species. Between the analyzed *Hilaria* species there was an observed pairwise distance of ca. 9–17%; in *Empis* species the distances were of 14–18% (see Table 2 and Table 3). We do not presume to evaluate any phylogenetic relationships based on a single gene. However, based on the COI gene marker of the chosen European species, the closest to the newly described *Empis sanrossorensis* sp. nov. could be *E. bicuspidata*; and to *H. polymorpha* sp. nov., it could be both *Hilara sericata* and *H*. *nigritarsis* species.

During the analyses, we also discovered some discrepancies in DNA sequences assigned to certain taxa. Specifically, the two *H. cornicula* specimens (MN868950 and OK065536) are most probably misidentified because of their very high pairwise distance value, which signals an interspecific distance range (12.5%). The opposite situation occurred in analyzed *Microphor* specimens assigned to *M. holosericeus* (MZ631891) and *M. crassipes* (MG823460), respectively, where the pairwise distance was of 0.02%, which signals the affiliation to the same species.

So, as we stated in the beginning, we consider the DNA evidence of the newly described species as of very high importance for further analyses and valuable contribution to the knowledge of the taxa, and this process should be performed carefully and with collaboration with a taxa-specialist.

## Figures and Tables

**Table 1:** List of analyzed specimens. Sequences obtained for this study in italics.

Taxa	Author and Year	GB Access No.	Locality
*Dolichopus stenhammari*	Zetterstedt, 1843	MG472184	Canada
*Dolichopus lamellipes*	Walker, 1849	MG476941	Canada
*Empis bicuspidata*	Collin, 1927	MN868986	Sweden
*Empis lucida*	Zetterstedt, 1838	MN868972	Sweden
*Empis nuntia*	Meigen, 1838	MN868989	Sweden
*Empis sanrossorensis_*male	Barták, sp. nov.	PQ344943	Italy
*Empis sanrossorensis_*female	Barták, sp. nov.	PQ344944	Italy
*Empis stercorea*	Linnaeus, 1761	MN868981	Sweden
*Hilara caucasica*	Kustov, Shamshev and Grootaert, 2013	KC589433	Russia
*Hilara cornicula_*1	Loew, 1873	MN868950	Sweden
*Hilara cornicula_*2	Loew, 1873	OK065536	Finland
*Hilara griseola*	Zetterstedt, 1838	KF297866	Norway
*Hilara interstincta*	Meigen, 1838	KF297874	Norway
*Hilara lapponica*	Chvála, 2002	OK065516	Finland
*Hilara nigritarsis*	Zetterstedt, 1838	KF297867	Norway
*Hilara obscura*	Meigen, 1822	KP264788	?
*Hilara polymorpha_*1	Barták, sp. nov.	PQ344941	Italy
*Hilara polymorpha_*2	Barták, sp. nov.	PQ344942	Italy
*Hilara pseguashae*	Kustov, Shamshev and Grootaert, 2013	KC589435	Russia
*Hilara seriata*	Loew, 1864	KP264789	?
*Hilara tanythrix*	Frey, 1913	KF297853	Norway
*Microphor crassipes*	Macquart, 1828	MG823460	?
*Microphor holosericeus*	(Meigen, 1804)	MZ631891	Finland
*Rhamphomyia anaxo*	Walker, 1849	JF879601	Canada
*Rhamphomyia calimenda*	Barták, 2002	KP264882	?
*Rhamphomyia clavigera*	Loew, 1861	JN302521	Canada
*Rhamphomyia erythrophthalma*	Meigen, 1830	MN868965	Sweden

**Table 2:** Pairwise distances computed using K2P correction between analyzed *Empis* species.

Taxa	GB Access No.	1	2	3	4	5	6
*Empis sanrossorensis*_male	PQ344943						
*Empis sanrossorensis*_female	PQ344944	0.002					
*Empis stercorea*	MN868981	0.183	0.183				
*Empis nuntia*	MN868989	0.175	0.178	0.169			
*Empis lucida*	MN868972	0.159	0.159	0.155	0.173		
*Empis bicuspidata*	MN868986	0.147	0.147	0.149	0.154	0.173	

**Table 3:** Pairwise distances computed using K2P between analyzed *Hilaria* species.

Taxa	GB Access No.	1	2	3	4	5	6	7	8	9	10	11	12	13
*Hilara polymorpha_*01	PQ344941													
*Hilara polymorpha_*02	PQ344942	0.009												
*Hilara pseguashae*	KC589435	0.118	0.123											
*Hilara caucasica*	KC589433	0.119	0.127	0.158										
*Hilara cornicula*	MN868950	0.126	0.137	0.139	0.156									
*Hilara seriata*	KP264789	0.088	0.097	0.125	0.125	0.127								
*Hilara obscura*	KP264788	0.126	0.136	0.164	0.170	0.150	0.141							
*Hilara interstincta*	KF297874	0.098	0.108	0.136	0.155	0.136	0.118	0.114						
*Hilara lapponica*	OK065516	0.105	0.115	0.132	0.144	0.150	0.124	0.152	0.126					
*Hilara cornicula* 2	OK065536	0.127	0.132	0.145	0.152	0.118	0.168	0.158	0.140	0.156				
*Hilara griseola*	KF297866	0.122	0.127	0.127	0.156	0.143	0.117	0.147	0.129	0.127	0.139			
*Hilara nigritarsis*	KF297867	0.093	0.102	0.122	0.115	0.145	0.121	0.133	0.124	0.134	0.138	0.138		
*Hilara tanythrix*	KF297853	0.109	0.114	0.136	0.113	0.156	0.121	0.130	0.124	0.140	0.139	0.132	0.110	

## Data Availability

Specimens used in this study are shared based on request from the CULSP collection.
